# Pericardial Effusion as the Initial Presentation of Systemic Lupus Erythematosus in a 37-Year-Old Female

**DOI:** 10.7759/cureus.49080

**Published:** 2023-11-19

**Authors:** Ano Shalomi, Ramanathan Ramesh

**Affiliations:** 1 General Medicine, Teaching Hospital Batticaloa, Batticaloa, LKA

**Keywords:** pericardiostomy, hypoalbuminemia, microscopic colitis, systemic lupus erythematosus, pericardial effusion

## Abstract

Systemic lupus erythematosus (SLE) is a complex autoimmune disease that can affect various organs and systems in the body, leading to a wide range of clinical manifestations. Pericardial effusion, which is an accumulation of excessive fluid in the pericardial sac surrounding the heart, can be one of the early presentations of SLE in some individuals. When it occurs in young females, it can be particularly concerning, as SLE predominantly affects women of childbearing age.

In this case report, we describe pericardial effusion as the initial presentation of SLE in a 37-year-old mother of four children. Importantly, early diagnosis and consistent follow-up are critical for improving the prognosis and quality of life for individuals with SLE.

## Introduction

Although SLE can affect most organs in the body, cardiac involvement frequently occurs [1,2]. It can affect all components of the heart, including the pericardium, myocardium, endocardium, valves, coronary arteries, and the conductive system. Pericardial involvement is common among cardiac manifestations, but presenting with pericardial effusion as the initial presentation is rarely reported.

Pericardial effusion can result from various causes, such as infection (viral, bacterial, and tuberculous), inflammation, metabolic conditions like uremia and hypothyroidism, malignancy, trauma, and autoimmune diseases like SLE and rheumatoid arthritis [3]. It can be the result of acute accumulation or chronic pericardial effusion. Chronic accumulation frequently presents due to autoimmune conditions, malignancy, or idiopathy.

Pericardial effusion may present with nonspecific symptoms such as central chest pain, dyspnea, fatigue, and tachycardia. It can be suspected based on physical examination findings like a shifted apex and muffled heart sounds, as well as from investigations such as an ECG showing low voltage complexes and chest X-ray showing cardiomegaly. The condition can be confirmed and the amount assessed with the help of a 2D echocardiogram or chest CT. The mainstay of treatment for pericardial effusion due to SLE is pericardiocentesis and the use of steroids. If recurrent pericardial effusion occurs, surgical intervention may be necessary.

There is no single test to diagnose SLE; it is classified with the help of clinical findings and investigation results using criteria developed by the European League Against Rheumatism (EULAR) and the American College of Rheumatology (ACR) [4]. In this case, we present a 37-year-old female patient with pericardial effusion classified as SLE according to the EULAR and ACR criteria.

## Case presentation

A 37-year-old female presented with a two-month history of exertional chest pain, exertional dyspnea, orthopnea, paroxysmal nocturnal dyspnea, and progressive bilateral lower limb edema. She denied having a history of fever, joint pain, oral ulcers, hair loss, or frothy urine. Previously, she was known to have recurrent sinusitis without obvious abnormalities on fiberoptic laryngoscopy. There was no family history of heart disease or autoimmune diseases. She was the mother of four children, with no history of miscarriages, and she denied a history of alcohol or illicit drug abuse.

At the time of admission to our hospital, she was afebrile, not pale, with a blood pressure of 120/70 mmHg, a pulse rate of 88 beats per minute, and oxygen saturation of 98% on room air. However, there was significant bilateral pitting lower limb edema. Her, precordial examination shows muffled heart sounds with a shifted apex. Her respiratory examination reveals a bilateral mild reduction in the intensity of breath sounds. There were no obvious positive findings in the abdominal examination.

**Figure 1:**
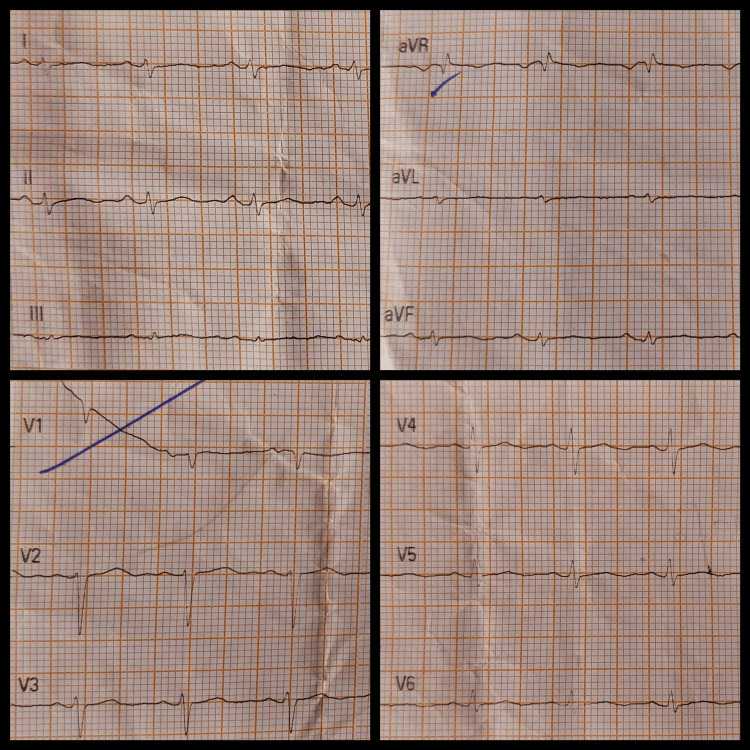
ECG shows low voltage QRS complex

**Figure 2:**
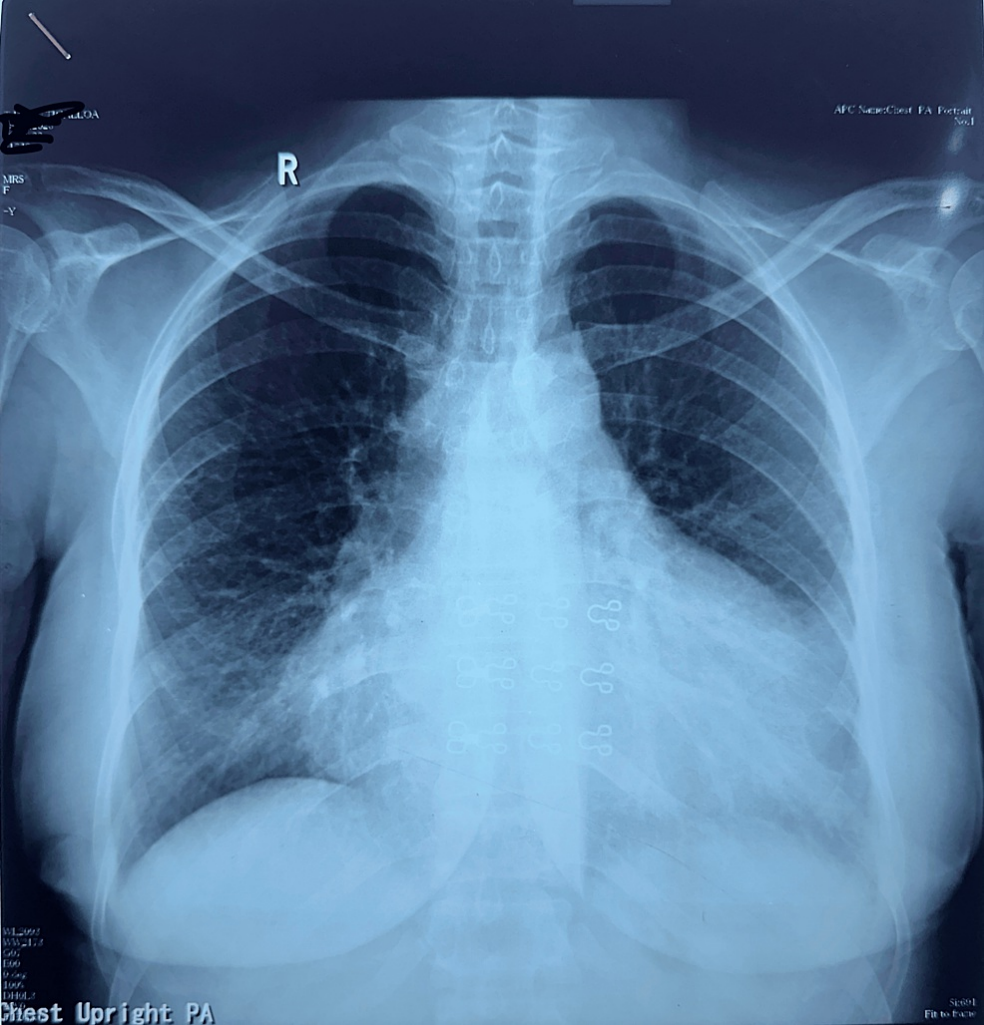
Chest X-ray shows cardiomegaly due to pericardial effusion

In the initial approach, we performed basic biochemical investigations and conducted an ECG, chest X-ray, and 2D echocardiogram. The ECG revealed a low-voltage QRS complex (Figure 1), and the chest X-ray showed cardiomegaly (Figure 2).

**Figure 3:**
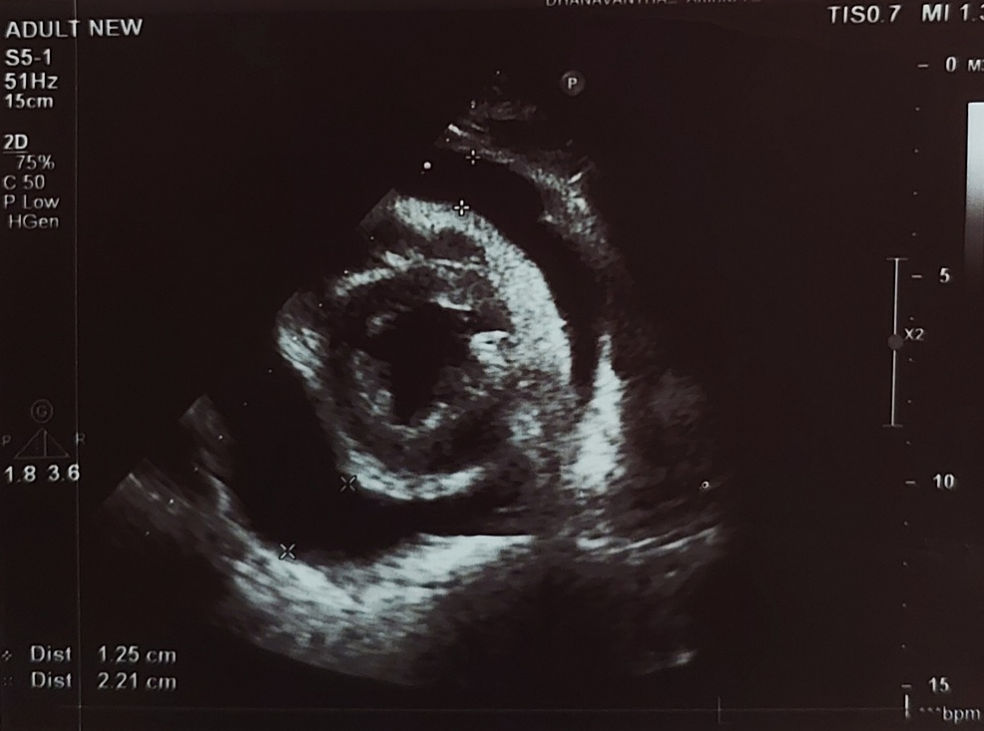
2D echocardiogram shows pericardial effusion

The 2D echocardiogram indicated an ejection fraction of 50% and severe, large pericardial effusion measuring 22mm anteriorly and 29mm posteriorly, without fibrin strands or signs of tamponade such as right atrial (RA) collapse, right ventricular (RV) collapse or diastolic variation (Figure 3). The patient immediately underwent pericardial aspiration due to a large pericardial effusion leading to impending tamponade by the cardiology team, during which 360ml of straw-colored fluid was aspirated and subsequently sent for further analysis.

At the same time, we investigated the etiology of the patient’s condition, which included auto-immune diseases, lymphoma, and tuberculosis. Surprisingly, the investigations related to autoimmune diseases, following the criteria of the European League Against Rheumatism (EULAR) and the American College of Rheumatology (ACR), led to the diagnosis of SLE. To further investigate the patient’s hypoalbuminemia, we offered an endoscopic examination to identify other features of SLE. The upper GI endoscopy revealed atrophied stomach mucosa, but the biopsy showed normal mucosa. In contrast, the lower GI endoscopy appeared normal, but the biopsy revealed microscopic colitis. Her routine investigations, investigations to find out etiology, and pericardial fluid analysis are plotted in Table 1.

**Table 1:** Investigations results during the ward stay. FBC: full blood count, WBC: white blood cell, AST: aspartate aminotransferase, ALT: alanine aminotransferase, PT: prothrombin time, INR: international normalized ratio, CRP: C-reactive protein, ESR: erythrocyte sedimentation ratio, FBS: fasting blood sugar, TSH: thyroid stimulating hormone, ADA: adenosine deaminase, LDH: lactate dehydrogenase, UA: uric acid, UFR: urine full report, UPCR: urine protein creatinine ratio, ANA: anti-nuclear antibody, dsDNA: double stranded DNA, USS: ultrasound scan, UGIE: upper GI endoscopy, LGIE: lower GI endoscopy.

Investigations		On admission	Reference range	
FBC	WBC	8.15 x 10^3^	4-11 x 10^3^	
	Neutrophil	5.15 x 10^3^	2-7 x 10^3^	
	Lymphocyte	1.98 x 10^3^	0.8-4 x 10^3^	
	Eosinophil	0.59 x 10^3^	0.02-0.05 x 10^3^	
	Hemoglobin	14.4	11-15 g/dL	
	Platelets	270 x 10^3^	150-450 x 10^3^	
Renal function Test	Blood urea	2.7	1.8-6.3 mmol/L	
	S. Creatinine	61	53-88 µmol/L	
Serum electrolytes	S. Sodium	142	136-145mmol/L	
	S. Potassium	3.7	3.5-5.1 mmol/L	
	S. Calcium	2.1	2.1-2.5 mmol/L	
	S. Magnesium	0.9	0.7-1.0 mmol/L	
	S. Phosphate	1.5	0.8-1.6 mmol/L	
Liver function Test	AST	22	15-37 U/L	
	ALT	25	12-78 U/L	
	ALP	55	46-116 U/L	
	Gamma -GT	21	15-85 U/L	
	T. Protein	57	64-82 g/L	
	S. Albumin	26	34-50 g/L	
	S. Globulin	31	22-48 g/L	
	T. Bilirubin	6.0	3.4-17.1 µmol/L	
	D. Bilirubin	1.2	0-3.4 µmol/L	
	PT/INR	1.37	<1.0	
Inflammatory markers	CRP	03	0-5 mg/l	
	ESR	15	<20mm/hr	
FBS	5.5		3.9-5.6 mmol/L	
Troponin I	<0.012		0-0.12	
Thyroid function test	TSH	6.21	0.46-4.68 mIU/L	
	T4	12.6	10.0-28.2 pmol/L	
Pericardial fluid analysis	Glucose	80mg/dl	40-80mg/dl	
	Protein	52 g/dL	30g/dl	
	Macroscopy	Turbid	Straw	
	Polymorphs	80/cumm	0	
	Lymphocytes	40/cumm	0	
	Red blood cells	1450/cumm	0	
	Culture	No growth		
	GeneXpert	Mycobacterium not detected		
	ADA	20	0-33 U/L	
	TB culture	Not detected		
Mantoux test	Negative			
Sputum AFB	3 samples negative			
LDH	372		81-234 U/L	
UA	277		155-357 µmol/L	
S. Ferritin	62.5		6.24-137 ng/mL	
UFR	Albumin	Trace	Nil	
	Pus cells	8-12	0-5	
	Red cells	nil	nil	
UPCR	45		>300-350 mg/mmol	
24 hours urine protein	182 mg/day		<150 mg/day	
Blood picture	No features of lymphoma, normal blood film.			
ANA	315		0-40 AU/mL	
ANA Titer	Positive		>1:80	
dsDNA	Positive (65.66)		>30 IU/mL	
Anti-Cardiolipin IgG Antibody	Positive (15.994)		>14.4	
Anti-Cardiolipin IgM Antibody	Negative (3.717)		>7.2	
Beta2 Glycoprotein IgG	9.4 (Equivocal)		>10 U/ml	
Beta2 Glycoprotein IgM	<2.9 (Negative)		>10 U/mL	
Lupus anticoagulant	0.94		0.8-1.2	
Complement C3	51.00 (low)		83-177 mg/dL	
Complement C4	13.00(Normal)		12-36 mg/dL	
USS abdomen	No intraabdominal mass or lymph nodes, no organomegaly, left side mild pleural effusion.			
UGIE biopsy	D2 mucosa: lamina propria shows a moderately lymphoplasmacytic infiltrate.no features of coeliac disease. Stomach body mucosa: appearances are those of reactive gastropathy, negative for neoplasm.			
LGIE biopsy	Lamina propria show a moderate lymphocytic infiltrate, granuloma not seen, appearances are those of microscopic colitis.			

We initially began treatment with steroids and hydroxychloroquine. The patient’s lower limb edema and hypoalbuminemia showed improvement [5]. However, there was a recurrence of pericardial fluid accumulation, requiring weekly aspirations for nearly three weeks. At this point, we considered the condition refractory and decided to refer the patient to the cardiothoracic unit for a pericardiostomy procedure.

The patient underwent pericardiostomy, and the histological examination of the pericardial tissue revealed a patchy mild lymphohistiocytic infiltrate. Granulomas were not present, and no tumors were detected. Following the pericardiostomy procedure, the patient became free from cardiac symptoms, and we arranged for regular follow-up appointments to monitor her disease condition.

## Discussion

This case serves as a reminder that there are numerous potential causes of pericardial effusion, and it can sometimes be the initial presentation of an autoimmune disease like systemic lupus erythematosus (SLE). It is important to keep this in mind when evaluating patients who present with pericardial effusion.

In our patient, initially, we suspected heart failure based on the history, but the examination raised suspicions of pericardial effusion. Subsequently, an echocardiogram confirmed the diagnosis of pericardial effusion, and further investigations revealed hypoalbuminemia. We conducted parallel investigations to determine the etiology, including sputum analysis, Mantoux test, GeneXpert, and Tuberculosis culture, all of which returned negative results, effectively excluding tuberculosis as a cause of the effusion. Additionally, the patient’s blood tests and ultrasound did not indicate any signs of lymphoma. 

Therefore, we shifted our focus towards assessing autoimmune diseases as a potential underlying cause. Based on the antibodies panel and clinical manifestations, we plotted against the EULAR and ACR criteria. The patient’s score reached 16 points, which exceeds the threshold of 10 points necessary for classification as SLE. Consequently, we diagnosed our patient with SLE.

During the investigation for hypoalbuminemia, we explore potential causes. However, urine evaluation did not provide evidence to explain the significantly low albumin levels. The patient denied reduced nutritional intake or diarrhea, even though we offered an endoscopic examination. Subsequently, the lower GI endoscopy revealed normal mucosa, but serial biopsies demonstrated features of lymphocytic infiltrate and microscopic colitis. These findings further supported our diagnosis of SLE, as such manifestations can be associated with this autoimmune condition [6].

In our patient, the microscopic colitis showed improvement with steroid therapy, as evidenced by an increase in the albumin levels. Additionally, the bilateral lower limb edema reduced over time. However, the pericardial effusion did not respond fully to medical management, and as a result, it required surgical pericardiostomy to achieve a full resolution of cardiac symptoms.

The patient presented with pericardial effusion as the initial manifestation of systemic lupus erythematosus (SLE), as previously reported in case reports and studies [7]. The treatment options for such cases typically include steroids, drainage, and surgical intervention for refractory cases. Early diagnosis and treatment have a significant impact on reducing mortality and morbidity [8].

## Conclusions

While cardiac manifestations are common in SLE, an initial presentation as pericardial effusion is rare. Therefore, when evaluating patients with pericardial effusion, it is essential to consider autoimmune diseases as a potential differential diagnosis, as it could be the initial presentation of autoimmune diseases such as SLE. Early diagnosis and treatment play a crucial role in improving the patient’s quality of life. While some cases of pericardial effusion due to SLE can fully respond to medical treatment, it’s important to note that refractory cases may require surgical pericardiostomy for resolution.
